# Antioxidant Treatment Alters Peripheral Vascular Dysfunction Induced by Postnatal Glucocorticoid Therapy in Rats

**DOI:** 10.1371/journal.pone.0009250

**Published:** 2010-02-17

**Authors:** Emilio A. Herrera, Misha M. Verkerk, Jan B. Derks, Dino A. Giussani

**Affiliations:** 1 Department of Physiology, Development, and Neuroscience, University of Cambridge, Cambridge, United Kingdom; 2 Department of Perinatology, University Medical Centre Utrecht, Utrecht, The Netherlands; University of Tor Vergata, Italy

## Abstract

**Background:**

Postnatal glucocorticoid therapy in premature infants diminishes chronic lung disease, but it also increases the risk of hypertension in adulthood. Since glucocorticoid excess leads to overproduction of free radicals and endothelial dysfunction, this study tested the hypothesis that adverse effects on cardiovascular function of postnatal glucocorticoids are secondary to oxidative stress. Therefore, combined postnatal treatment of glucocorticoids with antioxidants may diminish unwanted effects.

**Methodology/Principal Findings:**

Male rat pups received a course of dexamethasone (Dex), or Dex with vitamins C and E (DexCE), on postnatal days 1–6 (P1–6). Controls received vehicle (Ctrl) or vehicle with vitamins (CtrlCE). At P21, femoral vascular reactivity was determined via wire myography. Dex, but not DexCE or CtrlCE, increased mortality relative to Ctrl (81.3 versus 96.9 versus 90.6 versus 100% survival, respectively; P<0.05). Constrictor responses to phenylephrine (PE) and thromboxane were enhanced in Dex relative to Ctrl (84.7±4.8 versus 67.5±5.7 and 132.7±4.9 versus 107.0±4.9% Kmax, respectively; P<0.05); effects that were diminished in DexCE (58.3±7.5 and 121.1±4.3% Kmax, respectively; P<0.05). Endothelium-dependent dilatation was depressed in Dex relative to Ctrl (115.3±11.9 versus 216.9±18.9, AUC; P<0.05); however, this effect was not restored in DexCE (68.3±8.3, AUC). Relative to Ctrl, CtrlCE alone diminished PE-induced constriction (43.4±3.7% Kmax) and the endothelium-dependent dilatation (74.7±8.7 AUC; P<0.05).

**Conclusions/Significance:**

Treatment of newborn rats with dexamethasone has detrimental effects on survival and peripheral vasoconstrictor function. Coadministration of dexamethasone with antioxidant vitamins improves survival and partially restores vascular dysfunction. Antioxidant vitamins alone affect peripheral vascular function.

## Introduction

Glucocorticoids, such as dexamethasone, have been used to prevent or reduce the incidence of chronic lung disease [1], [2], an important cause of morbidity and mortality in premature infants [3], [4]. Their effectiveness is based on decreasing inflammatory responses, and accelerating lung maturation and surfactant production, thereby improving respiratory function in the infant. Hence, this clinical therapy has greatly decreased neonatal mortality and facilitated weaning from mechanical ventilation [2], [5].

Despite the well-established beneficial effects of postnatal glucocorticoid therapy, there has been serious concern regarding their clinical use because of unwanted side-effects. For instance, accumulating evidence shows that postnatal treatment with dexamethasone reduces somatic growth and weight gain in human infants [6], [7] and newborn rats [8], [9]. Postnatal dexamethasone treatment also induces hypertension and cardiomyopathy in premature babies [1], [2], [10] and in rat neonates [9], [11], [12], [13]. The mechanism underlying dexamethasone-induced hypertension may include glucocorticoid-induced increases in the sensitivity of the sympathetic nervous system [14], augmentation of vascular responses to other constrictor neurotransmitters and hormones [15], [16], and a diminished endothelium-dependent vasodilatation mediated through nitric oxide (NO), prostacyclin (PGI2) or the endothelium-derived hyperpolarizing factor (EDHF) [17], [18].

A separate line of evidence suggests that excess glucocorticoids promote oxidative stress, both by enhancing the production of reactive oxygen species (ROS) and/or by reducing the levels of endogenous antioxidants [19], [20]. As oxidative stress plays a major role in cardiovascular pathology, there is strong evidence that excessive ROS generation may underlie cardiovascular dysfunction related to glucocorticoids [19], [20].

In this study, we have tested the hypothesis that the unwanted side-effects of postnatal glucocorticoids on the developing cardiovascular system are also in part due to glucocorticoid-induced oxidative stress. If true, combined treatment of premature infants with glucocorticoid and antioxidants may ameliorate the unwanted side-effects while maintaining the beneficial effects of glucocorticoid therapy in the postnatal period. The hypothesis was tested by investigating in rats the effects on peripheral vascular reactivity at weaning of postnatal dexamethasone therapy with or without vitamins C and E.

## Results

### Body Weight and Survival

Pups were all born spontaneously on day 22 of gestation with no difference in birth weight (Table 1). Relative to controls, treatment with dexamethasone markedly reduced survival. In marked contrast, co-administration of vitamins C and E with dexamethasone significantly improved pup survival (Table 1). The increased mortality in dexamethasone treated pups was related with a higher prevalence of observed peritonitis (defined as distended and red abdomen with diffuse abdominal rigidity) and diarrhoea (yellow secretions in perianal area), although these comparisons fell outside statistical significance (Table 1).

**Table 1:** Body weight and survival.

	Ctrl	Dex	DexCE	CtrlCE
**Birth weight** (g)	6.4±0.1	6.7±0.2	6.4±0.1	6.9±0.1
**Survival** (%)	100	81.3*	96.9	90.6
**Peritonitis** (ratio and %)	0/32 (0%)	11/32 (34.4%)	7/32 (21.9%)	1/32 (3.1%)
**Diarrhea** (ratio and %)	0/32 (0%)	13/32 (40.6%)	8/32 (25.0%)	0/32 (0%)
**P21 Body weight** (g)	55.42±0.67	49.71±0.74*	45.70±1.5*	60.50±0.67*
Eyes opening (d)	14.1±0.1	12.6±0.2*	12.2±0.3*	14.8±0.1*

Values are mean ± S.E.M for body weight at birth and postnatal day 21 (P21), the percentage of pups surviving, the prevalence of peritonitis or diarrhoea in pups following treatment, and the postnatal age at which the eyelids first opened. Groups are control (Ctrl), pups treated with dexamethasone (Dex), pups treated with dexamethasone combined with vitamins C and E (DexCE) and pups treated with vitamins C and E alone (CtrlCE). For data on birth weight, survival, peritonitis and diarrhea n = 32 for all groups; for data on P21 body weight: Ctrl, n = 32; Dex, n = 26; DexCE, n = 31; CtrlCE, n = 29; and for data on eyelids opening: Ctrl, n = 32; Dex, n = 26; DexCE, n = 31; CtrlCE, n = 30. Significant differences (P<0.05) are: *, vs Ctrl (Fisher's Exact Test for survival, One-Way ANOVA + Student Newman-Keuls test elsewhere).

At weaning, pups treated with dexamethasone with or without vitamins C and E were significantly lighter than control pups. By contrast, pups treated with vitamin C and E alone were significantly heavier than control pups (Table 1). Similarly, pups treated with dexamethasone with or without vitamins C and E opened their eyelids for the first time significantly earlier than control pups. By contrast, pups treated with vitamin C and E alone opened their eyelids for the first time significantly later than control pups (Table 1).

### Femoral Vasoconstrictor Function

**Figure 1:**
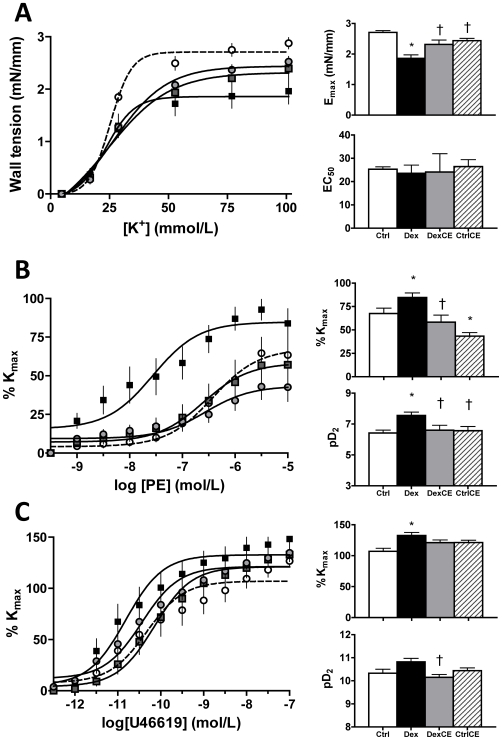
Vasoconstrictor function in pups at P21. Values are the mean ± S.E.M for the concentration-response curves, the maximal response (Emax or %Kmax) and the sensitivity (EC_50_ or pD_2_) to potassium (K^+^, A), phenylephrine (PE, B) and a thromboxane mimetic agent (U46619, C). Groups are control (Ctrl, open circle/histogram, n = 8), pups treated with dexamethasone (Dex, closed square/histogram, n = 8), pups treated with dexamethasone combined with vitamins C and E (DexCE, grey square/histogram, n = 8) and pups treated with vitamins C and E alone (CtrlCE, grey circle and hatched histogram, n = 8). Emax is the maximal effective tension induced by potassium-depolarization and % Kmax is expressed as the percentage of the maximal vasoconstriction induced by 76.88mM potassium. EC_50_ is the half maximal effective concentration and pD_2_ is –log(EC_50_). Significant differences (P<0.05) are: * vs Ctrl, † vs Dex (One-Way ANOVA + Student Newman-Keuls Test).

In pups treated with dexamethasone the maximal contraction (Emax) to K^+^ was significantly reduced relative to controls. In contrast, in pups treated with combined dexamethasone and vitamins C and E, the maximal constrictor response to K^+^ was restored towards control levels. Treatment of pups with vitamins C and E alone did not have an effect on the contractile response to K^+^. There were no differences in the vascular sensitivity to K^+^ across all four groups (Figure 1A).

Relative to controls, treatment of pups with dexamethasone increased the maximal contractile response (%Kmax) and the vascular sensitivity (pD_2_) to phenylephrine. Treatment of pups with combined dexamethasone and vitamins C and E restored towards control levels both the maximal constrictor response and the sensitivity to phenylephrine. Treatment of pups with vitamins C and E significantly reduced the maximal response but it did not affect the sensitivity to phenylephrine (Figure 1B).

Relative to controls, treatment of pups with dexamethasone increased the maximal contractile response (%Kmax) but not the vascular sensitivity (pD_2_) to the thromboxane mimetic agent. Treatment of pups with combined dexamethasone and vitamins C and E restored towards control levels the maximal constrictor response to the thromboxane mimetic agent. In addition, pups treated with combined dexamethasone and vitamins C and E had a significantly lower vascular sensitivity to the thromboxane mimetic agent than pups treated with dexamethasone. There was no effect on vascular reactivity to the thromboxane mimetic agent in pups treated with vitamins C and E alone (Figure 1C).

### Femoral Vasodilator Function

**Figure 2:**
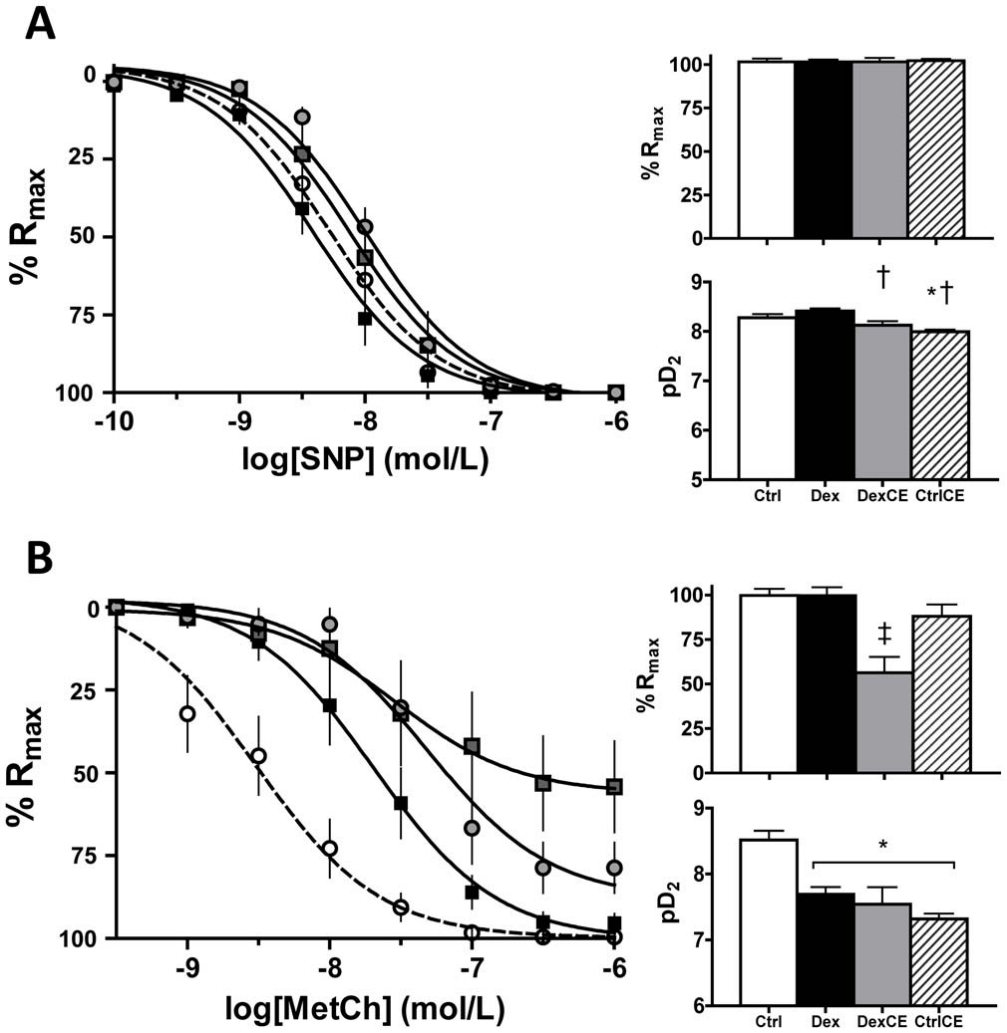
Vasodilator function in pups at P21. Values are the mean ± S.E.M for the concentration-response curves, the maximal relaxation (%Rmax) and the sensitivity (pD_2_) to sodium nitroprusside (SNP, A), and to methacholine (MetCh, B). Group numbers and group symbols are the same as in Figure 1. Significant differences (P<0.05) are: * vs Ctrl, † vs Dex, ‡ vs all (One-Way ANOVA + Student Newman-Keuls Test).

Endothelium-independent relaxation was assessed by generating cumulative concentration-response curves to the NO-donor sodium nitroprusside (SNP) following stable pre-contraction with 10^−5^ mol/L PE. Across all four groups, vessels showed complete relaxation to SNP, with no differences in Rmax. However, pups treated with combined dexamethasone and vitamins C and E had a lower sensitivity to SNP than pups treated with dexamethasone alone. Pups treated with vitamins C and E alone had a lower sensitivity to SNP than control pups (Figure 2A).

Endothelium-dependent relaxation was assessed by generating cumulative concentration response curves to methacholine (MetCh) after stable pre-contraction with 10^−5^ mol/L PE. Relative to controls, treatment of pups with dexamethasone or with vitamins, either in combination or alone, all significantly reduced the vascular sensitivity to MetCh. However, only pups treated with combined dexamethasone and vitamins C and E had a lower maximal relaxant response to MetCh relative to all groups (Figure 2B).

**Figure 3:**
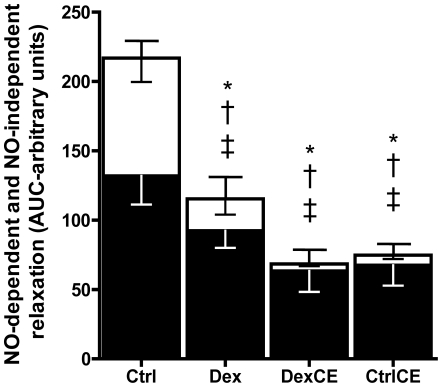
Partial contribution of NO-dependent and NO-independent mechanisms to the endothelial-dependent relaxation. Values are the mean ± S.E.M for the area under the curve (AUC) for MetCh-induced relaxation (complete bar with positive S.E.M.), the AUC for MetCh-induced relaxation following treatment with LNAME (NO-independent component, white bar with negative S.E.M.), and the remaining AUC after MetCh with LNAME (NO-dependent component, black bar with negative S.E.M). Group numbers are the same as in Figure 1. Significant differences P<0.05: * vs Ctrl for complete bar; † vs Ctrl for white bar, ‡ vs Ctrl for black bar (One-Way ANOVA + Student Newman-Keuls Test).

**Figure 4:**
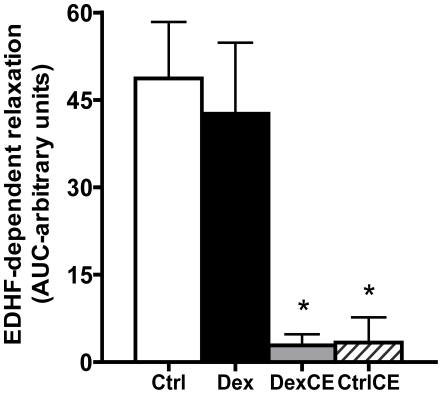
Partial contribution of EDHF to the NO-independent relaxation. Values are the mean ± S.E.M for the area under the curve (AUC) representing the relaxation mediated by the endothelium-derived hyperpolarizing factor (EDHF). The relaxant contribution mediated by EDHF is calculated by the AUC of MetCh-induced relaxation in the presence of LNAME and indomethacin. Groups are control (Ctrl, open histogram, n = 6), pups treated with dexamethasone (Dex, closed histogram, n = 6), pups treated with dexamethasone combined with vitamins C and E (DexCE, grey histogram, n = 6) and pups treated with vitamins C and E alone (CtrlCE, hatched histogram, n = 6). Significant differences (P<0.05) are: * vs Ctrl or Dex (One-Way ANOVA + Student Newman-Keuls Test).

Further analysis revealed that treatment of pups with dexamethasone or with vitamins, either in combination or alone, all significantly reduced the partial contributions of both NO-dependent and NO-independent mechanisms mediating the vascular relaxation (Figure 3). To dissect any effects of treatment on NO-independent mechanisms contributing to the vasomotor response induced by MetCh, the contribution of EDHF was determined. These data show that relative to controls the contribution of EDHF to the relaxant response induced by MetCh was maintained in pups treated with dexamethasone but it was significantly reduced in pups treated with vitamins C and E with or without dexamethasone (Figure 4).

## Discussion

Previous investigation of the effects of glucocorticoids on vascular function in the perinatal period have been restricted to the effects in the fetus [21], [22], [23], the newborn [24], [25] or in the adult offspring [26], [27] of antenatal treatment. This is the first study to determine the effects on vascular function of postnatal glucocorticoid therapy. The data show that: 1) clinically-relevant postnatal glucocorticoid therapy increases neonatal mortality, and augments vasoconstrictor while diminishing vasodilator reactivity in peripheral vessels isolated from weanling rats; 2) combined postnatal treatment of glucocorticoids with antioxidants improves neonatal survival and partially restores impairments in vascular reactivity; and 3) postnatal treatment with vitamins alone does not induce neonatal mortality or affect peripheral constrictor reactivity, but it diminishes peripheral vasodilator reactivity in weanling rats.

In addition to exogenous vasoactive influences such as neurotransmitters and hormones, and local factors such as those produced within the endothelium, it has become increasingly evident that the cellular oxidant *milieu* is also an important modulator of vascular resistance [28]. Vascular endothelial cells generate reactive oxygen species (ROS), such as the superoxide anion (^•^O_2_^−^) [29]. Superoxide readily combines with NO, limiting its bioavailability [30]. Hence, under physiological conditions, manipulation of the vascular NO∶^•^O_2_^−^ ratio is also an important determinant of tone. Data in the present study show that postnatal dexamethasone treatment diminished the contractile response to potassium, it increased the contractile responsiveness to α-adrenergic and local vasoconstrictors, and it depressed endothelium-dependent vasorelaxation. Postnatal treatment with combined dexamethasone and antioxidant vitamins restored contractile function, and postnatal treatment with vitamins alone diminished phenylephrine-induced contraction. The latter finding supports the concept that increased NO bioavailability acts to oppose tonic vasoconstrictor influences.

The depressive effects of dexamethasone on eNOS expression in a number of tissues are well described [31], [32]. In addition, dexamethasone may enhance ROS generation directly [19] or secondary to eNOS uncoupling by limiting the availability of cofactors such as tetrahydrobiopterin, BH4 [33]. Therefore, the increased vasoconstrictor responses in dexamethasone-treated pups in the present study may be due to glucocorticoid-induced blunting of tonic NO-dependent vasodilatation [15], [20]. Alternatively, the sensitizing effects of dexamethasone on vascular contractile function may be direct due to up-regulation of α-adrenoceptors in vascular smooth muscle cells (VSMCs), and/or increased intracellular uptake of calcium ions. For instance, perinatal dexamethasone is known to alter the pattern and expression of adrenoceptors in the rat [34] and sheep [35] and treatment of cultured VSMCs with dexamethasone for 48h increased expression of α_1B_ adrenoceptor mRNA [36]. Hayashi et al. [37] observed increased Ca^2+^ uptake and [3H]-dihydropyridine binding in VSMCs after a 48h dexamethasone incubation, suggesting that glucocorticoids may enhance VSMC contraction by inducing synthesis of new Ca^2+^ channels. The reversal of the sensitizing effects of dexamethasone on phenylephrine- and thromboxane-induced contractile function in pups treated with combined dexamethasone and antioxidants may be due to the effects of antioxidants restoring tonic NO bioavailaibility, replacing that which was diminished by dexamethasone. Alternatively, the reversal may be due to the effects of antioxidants increasing NO bioavailaibility, which act to oppose glucocorticoid-enhanced constrictor mechanisms.

The reduction in maximal tension induced by potassium in femoral vessels isolated from weanling rats treated with dexamethasone may indicate either a reduction in smooth muscle mass, or an impaired smooth muscle contraction, or both. Since glucocorticoids are known to inhibit the growth of vascular smooth muscle cells in culture [38] and to prevent neointimal vascular hyperplasia after injury in rats [39], it is plausible that a decrease in the smooth muscle∶extracellular matrix ratio may have produced the decreased responsiveness to potassium. Co-administration of dexamethasone with vitamins in the present study promoted recovery of the femoral contractile response to potassium. Given the lack of an effect of vitamins alone on potassium-induced contraction, it is unlikely that vitamins improve this response in dexamethasone-treated rats downstream of the increase in [Ca^2+^]_i_, such as by increasing myosin light chain kinase activity and/or the rate of cross-bridge cycling. Rather, the beneficial effects of antioxidant vitamins on potassium-induced contraction may again be due to correction of the oxidative imbalance within the vasculature of dexamethasone treated pups.

In the present study, pups treated with dexamethasone showed depressed vasodilator responses to metacholine, but not to SNP, indicating impairment of endogenous endothelium-derived vasodilators such as NO, EDHF and PGI_2_. The partial contributions of endogenous NO and EDHF to metacholine-induced vascular relaxation were further investigated following incubation of vessels with the NO synthase inhibitor L-NAME alone or after both L-NAME and the cyclooxygenase inhibitor indomethacin. These studies revealed that the dilator defect induced by dexamethasone was due to glucocorticoid-induced depression of both NO-dependent and NO-independent mechanisms. However, within NO-independent mechanisms, dexamethasone did not affect the contribution of EDHF, suggesting glucocorticoid-induced depression of prostanoid-dependent vasodilatation. Accordingly, it has been previously shown that both glucocorticoids and oxidative stress inhibit prostacyclin production [17], [30].

Postnatal treatment of pups with antioxidant vitamins alone or in combination with dexamethasone decreased NO-dependent vasodilator mechanisms, it almost abolished the contribution of EDHF to vasorelaxation, and it decreased femoral dilator reactivity to SNP. These effects may be due to negative feedback as a result of antioxidant vitamin-induced increases in NO bioavailability, which in turn may down-regulate the VSMC dilator pathway. For instance, chronic increases in NO have been reported to down-regulate sGC expression [40], [41] and to increase PDE function [42], thereby reducing SMC dilator capacity. Furthermore, inhibition of the NO-cGMP-PKG pathway will diminish VSMC relaxation due to lack of activation of calcium activated potassium channels [43], [44].

A final component of the data in the present study show that postnatal dexamethasone treatment increased neonatal mortality and accelerated maturational processes indexed by early eyelid opening, confirming previous obvervations in rat studies [9], [45], [46]. Interestingly, postnatal treatment with dexamethasone in combination with antioxidant vitamins did not prevent the maturational effects of glucocorticoids, but it markedly improved neonatal survival. Taken together, the data suggest that combined glucocorticoid and antioxidant treatment may diminish some of the adverse consequences of postnatal glucocorticoid therapy on mortality and on vascular function while maintaining maturational effects.

In conclusion, treatment of newborn rats with dexamethasone has detrimental effects on survival and peripheral vasoconstrictor function. Co-administration of dexamethasone with antioxidant vitamins improves survival and partially restores vascular dysfunction. Administration of vitamins alone to healthy pups decreases vasodilator capacity. The data support the hypothesis tested that unwanted side-effects of postnatal glucocorticoids on the developing cardiovascular system are in part due to glucocorticoid-induced oxidative stress, and that combined treatment of premature infants with glucocorticoid and antioxidants may ameliorate the unwanted side-effects while maintaining the beneficial effects of glucocorticoid therapy in the postnatal period.

## Materials and Methods

### Animals

All procedures were performed under the UK Animals (Scientific procedures) Act 1986 and were approved by the Ethical Review Committee of the University of Cambridge. Pregnant Wistar rats (n = 32; Charles River, UK) with timed gestations were individually housed under standard conditions (21°C room temperature/55% room humidity, light∶dark, 12∶12 hour) with *ad libitum* access to food (Special Diet Services, UK) and water. At birth (postnatal day 0, P0), each litter was sexed and the pups were weighed and culled to eight (four males/four females) to standardize feeding and maternal care within any one litter. All pups remained with their mothers until weaning at postnatal day 21 (P21).

### Postnatal Treatment Regimen

To exclude differences due to sex, only male rat pups were studied. The 4 male pups from each litter were randomly assigned to one of four treatments: Ctrl: n = 32; Dex: n = 32; DexCE: n = 32; CtrlCE: n = 32. Pups received two intraperitoneal (i.p.) injections per day (10 µL.g^−1^ each) of some or all of the following solutions (Sigma-Aldrich, UK): dexamethasone (Dexamethasone-21-phosphate, disodium salt), vitamin C (L-ascorbic acid), and vitamin E (dl-α-tocopherol acetate). Two injections were used due to the different solubility of vitamin E (dissolved in groundnut oil) and vitamin C and dexamethasone (in 0.9% NaCl). Ctrl pups received injections of the vehicles saline and groundnut oil for the duration of the treatment period (P1–6). Dex pups received a three-day, tapering course (0.5, 0.3, and 0.1 µg.g^−1^.day^−1^) plus separate injections of oil on P1–P3 and then only saline and oil from P4–P6. DexCE pups received the same treatment as Dex, plus vitamins C (200 mg.kg^−1^.day^−1^) and E (100 mg.kg^−1^.day^−1^) from P1–6. The CtrlCE pups received vitamins from P1–6. In our study, the dose and duration of the treatment was based on and is proportional to a 21-day tapering course of dexamethasone in humans. For extrapolation of postnatal treatment with dexamethasone in human neonates to postnatal treatment with dexamethasone in rat pups, the exposure to glucocorticoids has been estimated as a percentage of the total lactation period in the different species [11]. In humans, lactation averages 6 months. A 21-d tapering course of dexamethasone represents ca. 12% of the lactation period in humans. In rats, lactation is 21 days. 12% of the lactation period in rats is ca. 3 days. Doses of vitamins C and E used were adopted from studies indicating successful antioxidant effects in adult rats [47]. Ascorbic acid and α-tocopherol were combined as they act synergistically to provide optimal conditions for NO production [48].

### Wire Myography

At P21, to exclude differences due to within-litter variation, femoral arteries were isolated and collected from one male randomly chosen pup from each litter (Ctrl: n = 8; Dex: n = 8; DexCE: n = 8; CtrlCE: n = 8). These pups were humanely killed by CO_2_ inhalation and posterior cervical dislocation and their body weight (BW) was determined. Rats were kept on ice until vessel dissection. Under a bifocal dissecting microscope (Brunel Microscopes Ltd.), the first branch from the femoral artery of the left hind limb was excised via a medial incision. The vessel was placed in a Petri dish in phosphate-buffered saline kept on ice and carefully cleaned of adventitial, adipose and connective tissue. An arterial segment of approximately 2 mm length was cut and carefully threaded with a stainless steel wire (40 µm diameter). The threaded vessel segment was then placed between the mounting support jaws of a four-chamber small-vessel wire myograph (Multi Wire Myograph System 610M, DMT, Denmark). The wire was secured with screws to the jaw attached to the force transducer. A second wire was then passed through the arterial segment and its extremes fixed to the jaw attached to a micropositioner. The vessel segment was bathed in Krebs solution (mmol.l^−1^: NaCl 118.5, Fisher, KCl 4.75, Sigma, MgSO_4_•7H_2_0 1.2, Sigma, KH_2_PO_4_ 1.2, Sigma, NaHCO_3_ 25.0, Sigma, CaCl_2_ 2.5, Sigma, glucose 11.1, Sigma, UK) and gassed continuously with 5% CO_2_ and 95% O_2_ at 37°C in the myograph chamber.

Following a 15 minute equilibration period, the vessel was stretched in a stepwise manner to a standardized tension equivalent to a physiological transmural pressure [49]. This was done to simulate conditions *in vivo* for two main reasons; first, because the stimulated vascular response is dependent on the degree of stretch; and secondly, because this degree of stretch gives the maximal vascular response [50]. To test maximal contractile capacity, a potassium (K^+^) concentration-response curve was generated following a 20 minute equilibration period by subjecting vessels to increasing doses of a K^+^ solution (4.76mM to 100.94mM), washing with Krebs solution twice between doses. Cumulative concentration-response curves to the α1-adrenergic agonist phenylephrine (PE; 10^−10^–10^−5^ mol/L) and the thromboxane mimetic U46619 (10^−13^–10^−7^ mol/L) were determined in half-log increments. The relaxant effects of sodium nitroprusside (SNP; 10^−10^–10^−4^ mol/L) and methacholine (MetCh; 10^−10^–10^−4^ mol/L) were determined after pre-contraction with phenylephrine (10^−5^ mol/L). To determine the partial contributions of endogenous nitric oxide (NO) and EDHF to MetCh-induced vascular relaxation, additional concentration-response curves to MetCh were also determined after incubation with either L-NAME (10^−5^ mol/L) alone or after both L-NAME and indomethacin (10^−6^ mol/L). Between experiments, vessels were washed repeatedly with Krebs solution and allowed to equilibrate in Krebs solution for at least 20 minutes.

### Data and Statistical Analyses

The vascular response to potassium was analyzed using the Boltzmann sigmoidal analysis, and the maximal effective tension (Emax) and the half maximal effective concentration (EC_50_) were determined. All other concentration-response curves were analyzed using an agonist-response best-fit line, where the maximal vasomotor response was expressed as percentage of the contraction induced by 76.88 mM K^+^ (%Kmax for constriction, %Rmax for relaxation) and the vascular sensitivity was expressed as pD_2_(−logEC_50_).

Differences in the vascular responses were compared by calculating the area under the curve using the following equation:
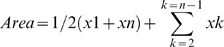
Where x is the response variable, n is the number of dose intervals during the protocol and xk is the response in the k^th^ interval.

The contribution of NO to the vascular relaxation induced by MetCh was calculated by subtracting the area under the curve (AUC) for MetCh – the AUC for MetCh + LNAME. The contribution of NO-independent mechanisms to the vascular relaxation induced by MetCh was calculated by the AUC for MetCh + LNAME. The EDHF dependent relaxation was calculated by the AUC after MetCh + LNAME & indomethacin.

Data are presented as mean ± S.E.M unless otherwise stated. Ratios and percentages were arcsine transformed prior to statistical analysis. Survival data were compared statistically using Fisher's Exact Test. Other data were compared statistically by One-Way ANOVA followed by the Student Newman-Keuls post hoc test. Significance was accepted when P<0.05 (SigmaStat 2.0; SPSS Inc., Chicago, USA).
